# Correction: Ponzo et al. Nucleolin Therapeutic Targeting Decreases Pancreatic Cancer Immunosuppression. *Cancers* 2022*, 14*, 4265

**DOI:** 10.3390/cancers14246160

**Published:** 2022-12-14

**Authors:** Matteo Ponzo, Anais Debesset, Mélissande Cossutta, Mounira Chalabi-Dchar, Claire Houppe, Caroline Pilon, Alba Nicolas-Boluda, Sylvain Meunier, Fabio Raineri, Allan Thiolat, Rémy Nicolle, Federica Maione, Serena Brundu, Carina Florina Cojocaru, Philippe Bouvet, Corinne Bousquet, Florence Gazeau, Christophe Tournigand, José Courty, Enrico Giraudo, José L. Cohen, Ilaria Cascone

**Affiliations:** 1 Immune Regulation and Biotherapy, Inserm U955, IMRB University of Paris-Est Creteil (UPEC) 8, INSERM, IMRB, F-94010 Créteil, France; 2 Cancer Research Center of Lyon, Cancer Cell Plasticity Department, University of Lyon, UMR INSERM 1052 CNRS 5286, Centre Léon Bérard, F-69008 Lyon, France; 3 AP-HP, Groupe Hospitalo-Universitaire Chenevier Mondor, Centre D’investigation Clinique Biothérapie, F-94010 Créteil, France; 4 Matières et Systèmes Complexes (MSC), Université de Paris, CNRS UMR 7057, F-75006 Paris, France; 5 Programme Cartes d’Identité des Tumeurs (CIT), Ligue Nationale Contre le Cancer, F-75013 Paris, France; 6 Laboratory of Tumor Microenvironment, Candiolo Cancer Institute, FPO-IRCCS, 10060 Candiolo, Italy; 7 Department of Science and Drug Technology, University of Torino, 10125 Torino, Italy; 8 Ecole Normale Supérieure de Lyon, University of Lyon, F-69342 Lyon, France; 9 UMR INSERM-1037, Cancer Research Center of Toulouse (CRCT), Toulouse University III, F-31037 Toulouse, France; 10 AP-HP, Service d’Oncologie Médicale, Groupe Hospitalo-Universitaire Chenevier Mondor, F-94010 Créteil, France

## Error in Figures

In the original publication [1], there was a mistake in Figure 3A, Figure 6E and Figure S1A: there are white squares in the picture which cover the area that the authors want to indicate.

The correct figures are attached below:

**Figure 3:**
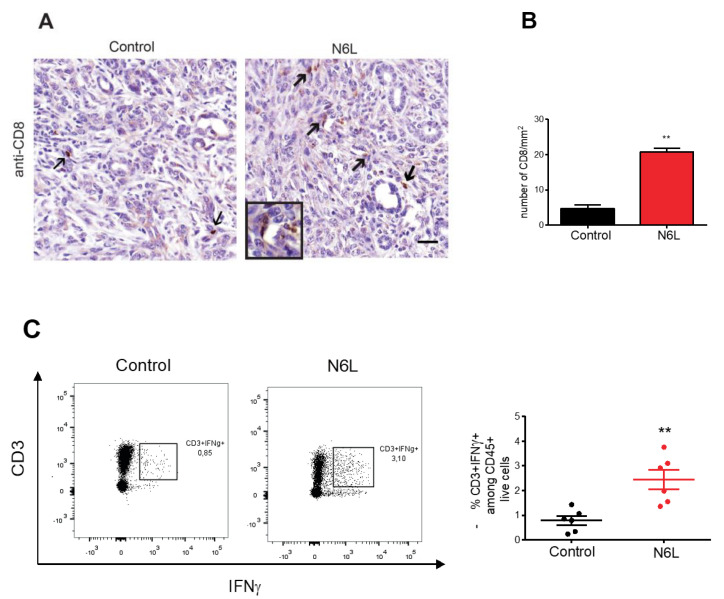
N6L increases lymphocyte infiltration and activation in mPDAC tumours. (**A**) Tumour sections of control and N6L-treated mice were immunostained by an anti-CD8 antibody to detect CD8^+^ cells (arrows); scale bar: 50 μm. (**B**) CD8^+^ cells in tumoral regions (at least 4) of tumour slices were counted by using QuPath software, and the results were plotted as a mean for each tumour (n = 6) as the number of cells/mm^2^. Two-tailed Mann-Whitney U-test (**, *p* < 0.01; n = 5 tumours) was applied. (**C**) Effector T cell activation was analysed by flow cytometry, and the % of CD3^+^IFNγ^+^ cells in control and N6L-treated mice is plotted (two-tailed Mann-Whitney U-test **, *p* < 0.01; n = 6 mice).

**Figure 6:**
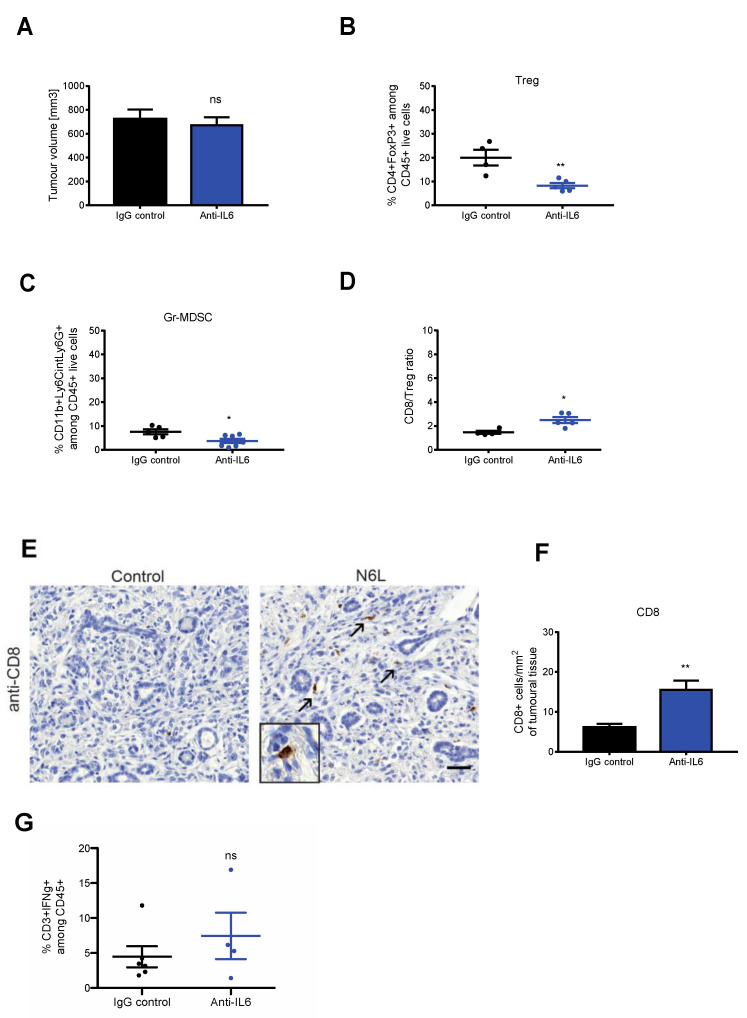
Anti-IL-6 antibody mimics N6L effects on PDAC immune microenvironment. mPDAC tumours were generated as in Figure 2 and treated with anti-IL-6 blocking antibody or IgG control antibody for three weeks, three times per week, by i.p. injection. Mice were sacrificed 21 days after the treatments. (**A**) Tumour volumes were measured as in Figure 2, and graphs show a representative experiment (from two independent experiments). (**B**–**D**) Immune cell populations in tumour tissues were analysed by flow cytometry as in Figure 2. Tregs and PMN-MDSC were analysed, and the results are plotted as the fold change of the % of (**B**) CD45^+^CD4^+^FoxP3^+^ cells and (**C**) CD11b^+^Ly6G^+^Ly6C^low^ cells in N6L-treated tumours relative to control tumours. (**D**) Fold change of the CD8/Treg ratio between control and N6L-treated tumours. (**E**) Tumour sections of control and anti-IL-6-treated mice were immunostained by an anti-CD8 antibody, CD8^+^ cells were counted (arrows), and results were plotted as (**F**) the number of cells/mm^2^. Scale bar: 50 μm. *p*-values were calculated between indicated conditions by two-tailed Mann-Whitney *t*-test (**, *p* < 0.01; *, *p* < 0.05; n = 5 mice). (**G**) Effector T cell activation was analysed by flow cytometry as in Figure 3C, and the % of CD3^+^IFNγ^+^ cells in control and N6L-treated mice is plotted (two-tailed Mann-Whitney U-test **, *p* < 0.01; control, n = 5 mice; anti-IL-6, n = 4 mice).

**Supplementary Figure S1:**
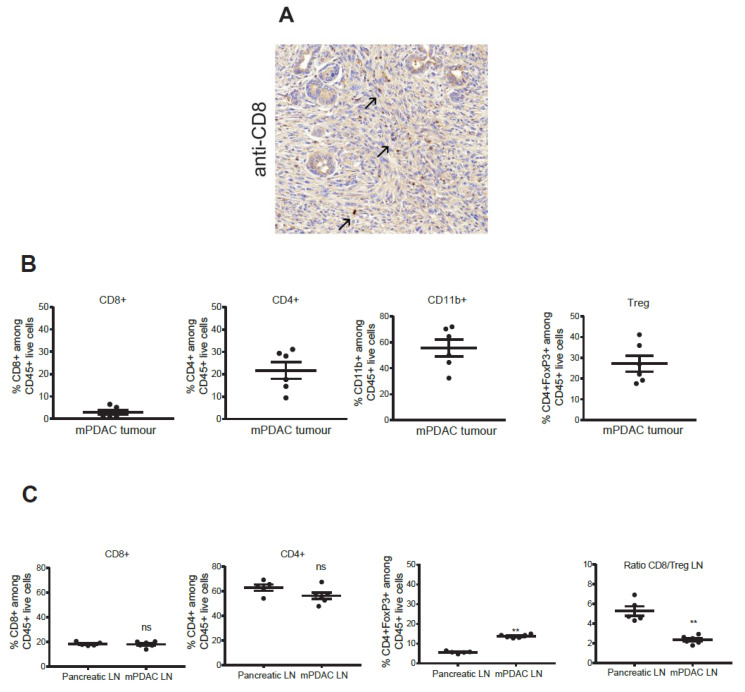
Characterization of mPDAC immune microenvironment. Immuno-competent syngenic FVB/n mice were injected with mPDAC cells into the pancreas. After three weeks, mice (n = 6) were sacrificed and tumours and draining lymph nodes were collected. (**A**) Tumour sections were immunostained an anti-CD45 antibody (arrows). (**B**,**C**) Immune cell populations of tumours and lymph nodes were analyzed by flow cytometry and frequency were calculated among CD45^+^ cells. Graphs show the % of CD45^+^CD8^+^, CD45^+^CD4^+^, CD45^+^CD11b^+^, CD45^+^CD4^+^FoxP3^+^ (Tregs among CD4^+^, and the ratio of CD8/Treg in tumours. All statistical tests are Two-tailed Mann-Whitney U-test; **, *p* < 0.01; ns = not significant, n = 6. Scale bars 50 μm.

The authors apologize for any inconvenience caused and state that the scientific conclusions are unaffected. The original publication has also been updated.
