# Supplementary material for: Nucleolin Therapeutic Targeting Decreases Pancreatic Cancer Immunosuppression
Source: Cancers (Basel). 2022 Aug 31;14(17):4265. doi: 10.3390/cancers14174265 (PMC9454580; doi:10.3390/cancers14174265)
Supplement: Supplementary file 1 [file cancers-14-04265-s001.zip › cancers-1822282-supplementary.pdf]

cancers MDPI

Supplementary Material: Nucleolin Therapeutic Targeting Decreases Pancreatic Cancer Immunosuppression

Matteo Ponzo, Anais Debesset, Mélissande Cossutta, Mounira Chalabi-Dchar, Claire Houppe, **Caroline Pilon, Alba Nicolas-Boluda, Sylvain Meunier, Fabio Raineri, Allan Thiolat, Rémy Nicolle, Federica Maione, Serena Brundu, Carina Florina Cojocaru, Philippe Bouvet, Corinne Bousquet, Florence Gazeau, Christophe Tournigand, José Courty, Enrico Giraudo, José L Cohen and Ilaria Cascone**

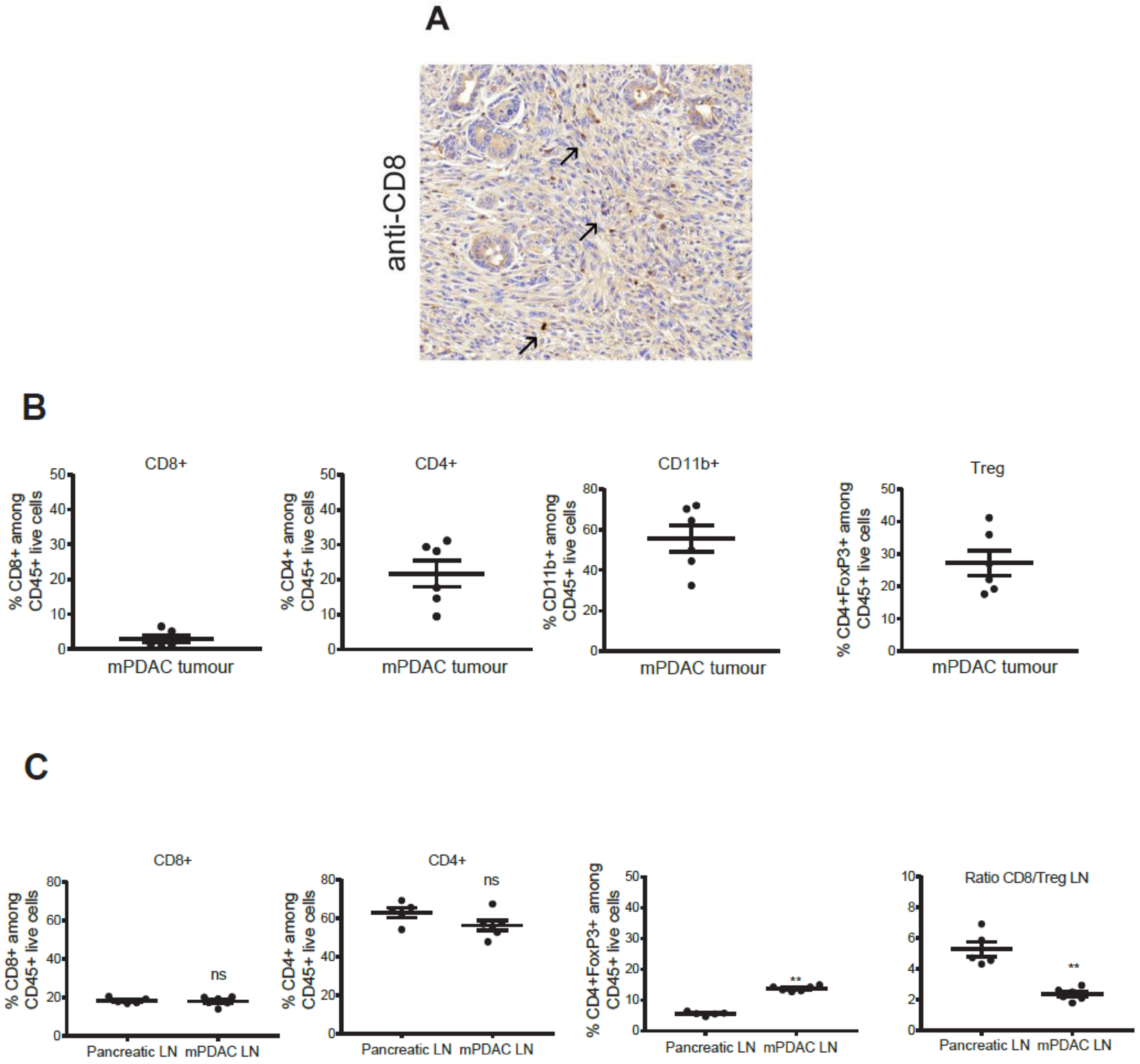

Supplementary Figure S1. Characterization of mPDAC immune microenvironment. Immunocompetent syngenic FVB/n mice were injected with mPDAC cells into the pancreas. After three weeks, mice (n = 6) were sacrificed and tumours and draining lymph nodes were collected. (**A**) Tumour sections were immunostained an anti-CD45 antibody (arrows). (**B**,**C**) Immune cell populations of tumours and lymph nodes were analyzed by flow cytometry and frequency were calculated among $CD45^+$ cells. Graphs show the % of $CD45^+CD8^+$, $CD45^+CD4^+$, $CD45^+CD11b^+$, $CD45^+CD4^+FoxP3^+$ (Tregs among $CD4^+$, and the ratio of CD8/Treg in tumours. All statistical tests are Two-tailed Mann-Whitney U-test; **, $p < 0.01$; ns = not significant, n = 6. Scale bars 50 μm.

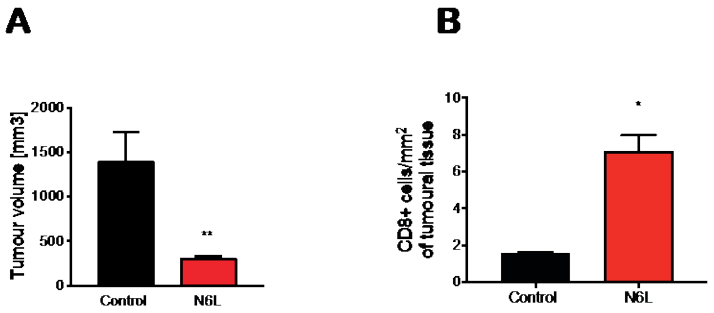

Supplementary Figure S2. N6L treatment of orthotopic KPC tumours. Immunocompetent syngenic C57BL/6 mice were injected with KPC cells into the pancreas. Mice were treated one week after cell inoculation with N6L alone (7 mg/kg) or saline solution by i.p. three times a week for five weeks. (**A**) Mice were sacrificed and tumour volumes were measured (two-tailed Mann-Whitney U-test, ** $p < 0.01$; n = 6 mice). (**B**) Tumour sections of control and N6L-treated mice were immunostained by an anti-CD8 antibody, and CD8$^+$ cells were counted in tumour regions (at least 4) as in Figure 3 and the mean of 5 tumours was plotted as number of cells/mm^2. Two-tailed Mann-Whitney U-test (** $p < 0.01$, n = 5 tumours) was applied.

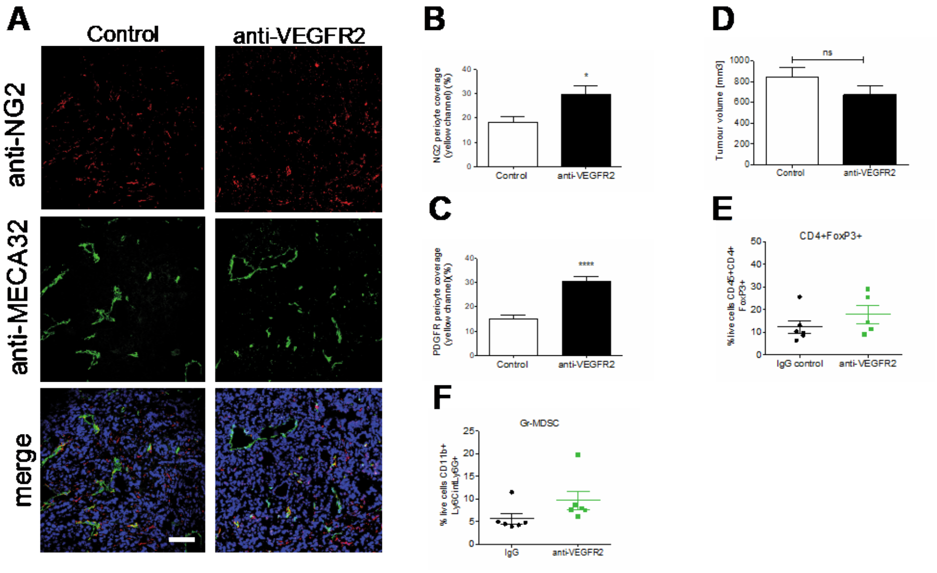

Supplementary Figure S3. VEGFR-2 inhibition in mPDAC models. Immunocompetent syngenic FVB/n mice were injected with mPDAC cells into the pancreas. Mice were treated one week after cell inoculation with IgG isotype control or anti-VEGFR-2 antibody by i.p. injections three times a week for three weeks. (**A**) Tumour sections of anti-VEGFR-2 treated mice or control mice were immunostained by an anti-MECA32 antibody that recognizes vessels (green), an anti-NG2 antibody that stains pericytes (red) and nuclei were stained with DAPI. (**B**,**C**) Pericyte coverage was calculated as the yellow merge between tumour vessels and pericytes as previously described (15), and graphs show the % of pericyte coverage for (**B**) NG2 positive pericytes or (**C**) PDGFR-◎ positive pericytes. (**D**) Mice were sacrificed, and tumour volumes were measured. (**E**,**F**) Immune cells infiltrated in tumours were analysed by flow cytometry and graphs show the % of (**E**) CD45$^+$CD4$^+$ FoxP3$^+$ cells and (**F**) PMN-MDSCs. P-values were calculated between indicated conditions by two-tailed Mann-Whitney U-test (****, $p < 0.001$; *, $p < 0.05$; n = 6).

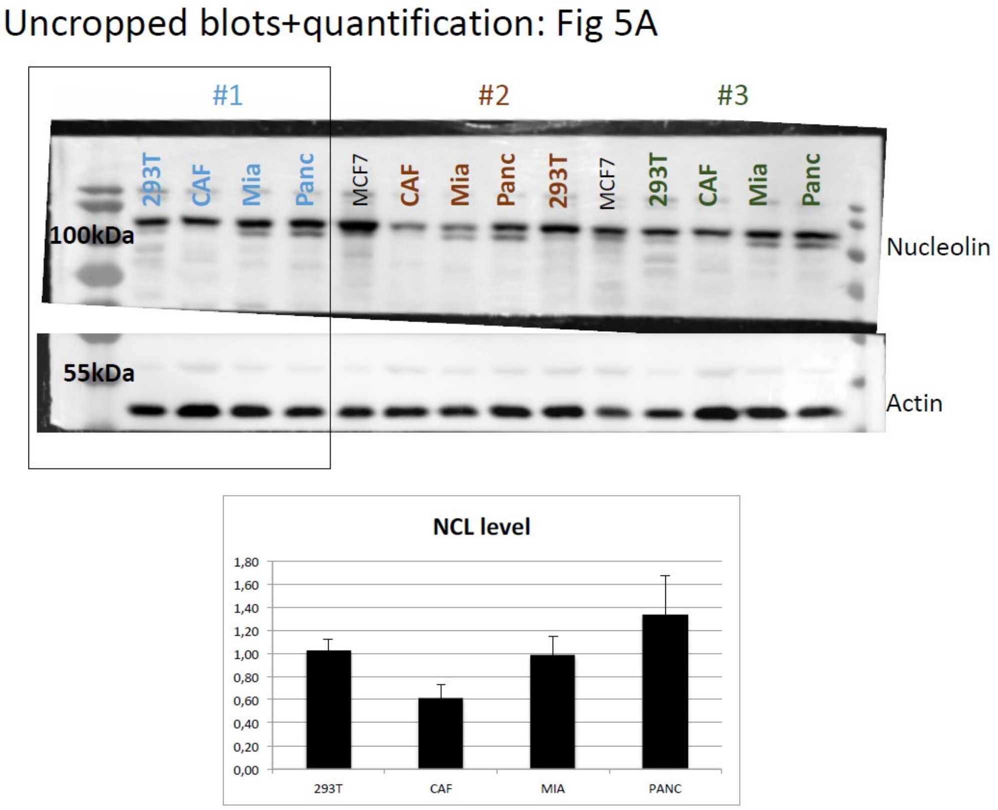

Supplementary Figure S4. uncropped Western Blot images.

Supplementary Table S1. List of the genes upregulated by more than 0.5-fold in N6L-treated tumours compared to control tumours by RNA-Seq analysis.

Top downregulated genes			
**Gene ID**	**log2FoldChange**	**pvalue.N6L-Control**	**padj.N6L-Control**
Ly6d	-1,38481726	1,05118E-08	4,34897E-05
Il1b	-1,171982702	9,83086E-07	0,001573862
Pkhd1l1	-1,128536965	3,82112E-06	0,002745574
Grem2	-1,123078207	2,75189E-07	0,000759017
Nlrp5-ps	-1,089233081	5,6028E-06	0,003090691
Tnc	-1,083031755	6,83376E-09	4,34897E-05
Calcb	-1,079876105	7,64779E-06	0,003616095
Hspa1b	-1,069479513	3,31207E-06	0,002745574
Mgl2	-1,057362243	6,44468E-06	0,003136854
Nr4a3	-1,044993194	6,21308E-06	0,003115767
Rian	-1,038481063	1,7871E-05	0,007436351
Il6	-1,025087173	2,73352E-05	0,009624912
Hspa1a	-1,023347985	1,22142E-05	0,005463047
Cyp2j5	-1,020926855	2,49525E-05	0,009384988
Cd200	-1,001740495	6,06993E-06	0,003115767

Supplementary Table S2. List of the genes downregulated by more than 0.5-fold in N6L-treated tumours compared to control tumours by RNA-Seq analysis.

Top upregulated genes			
**Gene ID**	**log2FoldChange**	**pvalue.N6L-Control**	**padj.N6L-Control**
Sema6b	0,478859994	8,47521E-05	0,021915035
Vars2	0,502996362	0,000119006	0,026075834
Phgdh	0,539561517	3,75756E-05	0,01243676
Rbm47	0,577514485	9,1407E-05	0,0222455
Ccdc14	0,578545578	0,000230307	0,042824119
AI661453	0,606730174	8,9655E-05	0,0222455
Rassf7	0,616811919	7,93215E-05	0,020836363
Cacna1d	0,630084557	0,000239696	0,0440747
Wbscr27	0,658816526	0,000216231	0,041609368
Ezr	0,668388261	5,21237E-06	0,003080695
Haghl	0,678807532	2,62179E-06	0,002520968
Lamc2	0,691830401	2,33074E-05	0,00914599
Hgfac	0,699480115	1,79741E-05	0,007436351
Coro2a	0,755117508	4,80464E-05	0,015290769
Krt19	0,759130706	1,14124E-06	0,001573862
Vstm5	0,787134367	2,65683E-05	0,00955823
Gldc	0,803441694	3,98174E-06	0,002745574
Cenpm	0,827387064	4,15455E-06	0,002750149
Qrfp	0,835105732	0,00017646	0,035183545
Cbs	0,83895381	6,97615E-07	0,001282758
Gclc	0,842585955	0,000251531	0,045742638
Metrn	0,851703863	9,50286E-09	4,34897E-05
Zbtb16	0,857890082	0,000254783	0,045830442
Gzmf	0,881278465	0,000285417	0,049934596
Chga	0,894906441	0,000175928	0,035183545
Kcnf1	0,896810522	0,000167492	0,034220165
Slc25a48	0,91491435	5,23882E-05	0,016357961
Cxcl13	0,923366972	0,0001114	0,025254262
Pcsk6	0,929387238	1,04944E-06	0,001573862
Cyp2s1	0,983122719	9,73405E-06	0,004474691
